# Calibration correction to improve registration during cone‐beam CT guided histotripsy

**DOI:** 10.1002/mp.17644

**Published:** 2025-01-26

**Authors:** Katrina L. Falk, Paul F. Laeseke, Grace M. Minesinger, Orhan G. Ozkan, Michael A. Speidel, Timothy J. Ziemlewicz, Fred T. Lee, Martin G. Wagner

**Affiliations:** ^1^ Department of Radiology University of Wisconsin‐Madison Madison Wisconsin USA; ^2^ Department of Biomedical Engineering University of Wisconsin‐Madison Madison Wisconsin USA; ^3^ Department of Medical Physics University of Wisconsin‐Madison Madison Wisconsin USA; ^4^ Department of Urology University of Wisconsin‐Madison Madison Wisconsin USA

**Keywords:** calibration, cone‐beam CT, histotripsy

## Abstract

**Background:**

Histotripsy is a non‐invasive, non‐ionizing, non‐thermal focused ultrasound technique. High amplitude short acoustic pulses converge to create high negative pressures that cavitate endogenous gas into a bubble cloud leading to mechanical tissue destruction. In the United States, histotripsy is approved to treat liver tumors under diagnostic ultrasound guidance but in initial clinical cases, some areas of the liver have not been treated due to bone or gas obstructing the acoustic window for targeting. To address this limitation in visualization, cone‐beam computed tomography (CBCT) guided histotripsy was developed to expand the number of tumors and patients that can be treated with histotripsy.

**Purpose:**

The purpose of this work is to improve the accuracy of CBCT guided histotripsy by calibrating the therapeutic bubble cloud location relative to the histotripsy robot arm.

**Methods:**

The calibration correction involves creating a bubble cloud sized treatment (a few mm) in an agar‐based phantom consisting of 11 layers with alternating high and low x‐ray attenuation. The layers were spaced ∼3 mm apart to allow visualization of mixing after mechanical disintegration from the histotripsy treatment. Bubble cloud treatments were localized using an automated algorithm that minimized a cost function based on the intensity difference within the treatment region on the pre‐ and post‐treatment CBCT. The actual treatment location can be compared to the theoretical bubble cloud location (focal point based on the CAD model of the transducer assembly) to calculate a 3D offset (*X*, *Y*, *Z*), which is used as the calibration correction between the therapeutic bubble cloud location and the histotripsy robot arm. The phantom and algorithm were analyzed to determine parameters that maximized bubble cloud treatment detection (treatment duration, localization accuracy of the phantom, number of bubble clouds) and were tested on four different histotripsy transducers.

**Results:**

Bubble cloud locations were accurately identified with the automated algorithm from post‐treatment CBCT images of the multilayer agar phantom. Treating the phantom for 20 seconds was associated with the greatest change in CBCT intensity. The phantom and algorithm were able to localize changes in bubble cloud location with mean residual errors (MRE) between the measured and planned translations of 0.3 ± 0.3 mm in *X*, −0.2 ± 0.6 mm in *Y*, and 0.1 ± 1.0 mm in *Z*. A multi‐bubble cloud calibration approach with four adjacent bubble clouds provided a statistically significant lower mean absolute deviation (MAD) in measured 3D offset (0.1, 0.0 and 0.2 mm in *X*, *Y*, and *Z*, respectively) compared to using a single bubble cloud (MAD of 0.2, 1.1 and 1.2 mm in *X*, *Y*, and *Z*, respectively). The calibration correction method measured statistically significantly different 3D transducer offsets between the four histotripsy transducers.

**Conclusions:**

Creating and analyzing four adjacent bubble clouds together produced more accurate and reproducible 3D offset measurements than analyzing individual bubble clouds. The presented histotripsy bubble cloud calibration correction method is automated, accurate, and can be easily integrated in the current histotripsy workflow to improve accuracy of CBCT guided histotripsy.

## 1 INTRODUCTION

Histotripsy is a non‐invasive, non‐ionizing, and non‐thermal tissue destruction technique that was recently FDA cleared to treat liver tumors (October 2023).^1^ The term “histotripsy” was first coined by the University of Michigan in 2004, with “histo” meaning soft tissue and “tripsy” meaning breakdown.^2^ The technology uses multiple ultrasound elements arranged in a hemispheric pattern to focus the ultrasound waves to a center point. The interference of the ultrasound waves at the focal point creates a mechanical disruption that leads to cavitation of nanoscale and microscale endogenous gas bubbles in the tissue. The repetitive expansion and contraction of the bubbles cause high shear stress and strain on the neighboring cells which lead to cell wall destruction and mechanical disintegration of the tissue.^3^, ^4^, ^5^ Cavitation cloud histotripsy (which was used in this study) uses microsecond long pulses (0.2–4 µs) at 250 kHz–6  MHz to generate a cloud of bubbles.^2^, ^6^, ^7^ During the first few pulses, bubbles are generated when the high peak negative pressure at the focal point exceeds the surface tension of the nanoscale gas pockets endogenous in tissue.^8^, ^9^ The pressure at which cavitation occurs is called the intrinsic cavitation threshold and varies per tissue (−25 to −28  MPa for water based tissue and −14 MPa for lipid‐based tissue).^6^, ^8^, ^10^ Then subsequent ultrasound pulses create shockwaves which increase the negative pressure in the area and create a cavitation ‘bubble cloud’ ranging in size from 3 × 3 × 7  mm to 4 × 4 × 10 mm.^11^, ^12^, ^13^ The reliance on the stability of the gas bubbles in their nano‐environment allows for tissue selective tissue destruction, which has been exemplified by liver treatments successfully destroying parenchyma but leaving bile ducts and blood vessels intact due to their higher collagen content.^10^, ^14^, ^15^, ^16^, ^17^ Histotripsy also has a low duty cycle which allows for any absorbed or frictional heat to dissipate before it reaches temperatures that create thermal damage (i.e., protein denaturation or coagulation necrosis).^18^, ^19^ The tissue selectivity and non‐thermal process make histotripsy advantageous over thermal ablation techniques (microwave ablation, radiofrequency ablation, or cryoablation) to treat in areas of the liver that are close to large vessels or bile ducts (i.e., liver hilum near central bile ducts).^20^, ^21^, ^22^, ^23^, ^24^, ^25^

The histotripsy “treatment zone” is created by translating the focal point (bubble cloud) through the pre‐planned volume under diagnostic ultrasound guidance. Because of the liver's anatomical location, some patients in the initial clinical trials had tumors that were not fully accessible for treatment due to bone or gas obstructing the diagnostic ultrasound targeting window.^26^ However, due to various physical factors such as lower frequency, higher amplitude, a singular direction and a specific transducer geometry, the therapeutic energy can still be delivered to tissue beyond the areas that diagnostic ultrasound can depict.^27^, ^28^ Therefore, C‐arm cone‐beam computed tomography (CBCT) was proposed for the guidance of histotripsy to overcome the limitation of diagnostic ultrasound and expand the number of tumors and patients that can be treated with histotripsy.^29^, ^30^

**FIGURE 1:**
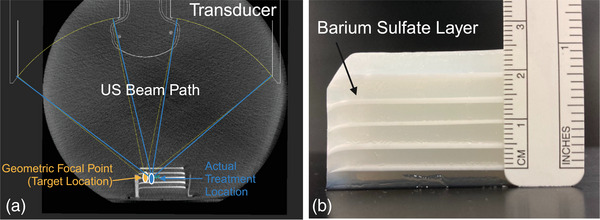
(a) Cone‐beam CT image of the phantom with alternating layers of x‐ray attenuation overlayed with the histotripsy transducer focal point. The geometric focal point is seen in yellow and an example of a possible experimentally measured focal point is in blue. Calibration in three directions of the actual treatment location (blue) to the histotripsy robot is necessary for accurate CBCT guided histotripsy treatments. (b) Image of the histotripsy phantom consisting of alternating layers with barium sulfate (opaque) and without (translucent), strategically placed ∼3–4 mm apart. Created in BioRender.^33^

CBCT guided histotripsy relies on spatial registration of the histotripsy transducer to the CBCT image volume and precise knowledge of the bubble cloud position relative to the transducer. The CBCT image‐to‐bubble cloud registration procedure estimates the transformations between (1) the coordinate systems of the CBCT imaging system and the robotic arm (image‐to‐robot registration) and (2) the coordinate systems of the robotic arm and the bubble cloud location. This was developed by Wagner et al in 2023 where a hand‐eye calibration method simultaneously estimated the transformations between coordinate systems of the CBCT volume, histotripsy robot, and bubble cloud location.^29^, ^30^ This method assumes the bubble cloud location is formed at the geometrical focal point of the transducer (determined by multi‐pose calibration or the CAD model), however, in practice the exact location of the bubble cloud may vary, for example, due to small variations in the transducer assembly or inaccuracies in the estimated transformation between robotic arm and transducer coordinate systems (Figure 1a). Therefore, a calibration correction is needed to account for this 3D deviation in bubble cloud location during the calibration step (offset). This offset, deemed “transducer offset”, is fundamentally different than any changes in location that arise from aberration from tissue speed of sound or attenuation.

Agar based phantoms have been used to visualize cavitation cloud histotripsy treatment zones in vitro with CBCT. Previous work has focused on creating phantoms for multimodality imaging^29^, ^31^ or assessment of the treatment zone over an agar target.^32^ In these studies, a full histotripsy treatment zone (2.0–2.5 cm) was needed for visualization, and manual segmentation was required for volume identification. Using a full treatment zone to measure precise calibration offsets of a bubble cloud could possibly confound results and therefore, a phantom that can visualize a single bubble cloud with high spatial accuracy is needed.

This work presents a method to calibrate the histotripsy robot to the therapeutic focal point by visualizing individual bubble cloud treatment zones in in vitro agar phantoms. This method identifies offsets between the theoretical and actual bubble cloud location to improve accuracy of CBCT‐guided histotripsy.

## 2 METHODS

### 2.1 Phantom creation

A 5 cm × 5 cm × 2.5 cm, multi‐layer phantom was created with layers of alternating high and low x‐ray attenuation. The phantom consists of agar (1.5%, Agar #12177, Fischer Scientific, St. Louis, MO) mixed with degassed, deionized water. Five layers are mixed with barium sulfate powder (6%, Barium Sulfate Powder, Carolina Biological Supply Company, Burlington, NC) to increase x‐ray attenuation, and six layers have no barium sulfate (plain). A concentration of 6% barium sulfate was used to ensure that visualization of the phantom through 20–30 L of water (the phantom needs to be positioned in a water bath for ultrasound coupling of the therapeutic energy) was possible.^31^ The agar solutions were prepared and degassed using a vacuum pump (2561B‐50 WOB‐L, Welch, Mt. Prospect, IL) and chamber (three gallon, BVV, Naperville, IL). An alternation of 8 mL of plain agar and 2 mL of agar with barium sulfate were placed into a silicone mold, allowing each layer to fully set in‐between. The layers were 3–4 mm apart to optimize visualization of the mixing between layers when mechanical disintegration occurs during histotripsy treatment. The phantoms were removed from the mold and placed in deionized water at low pressure in a vacuum chamber until treatment. Figure 1a shows a CBCT image of the phantom, illustrating the alternating layers of high and low x‐ray attenuation. The alternating layers are also seen in Figure 1b of the untreated phantom with layers of barium sulfate agar (white) and plain agar (opaque). The material cost to create each phantom is ∼$0.50.

### 2.2 CBCT guided histotripsy

**FIGURE 2:**
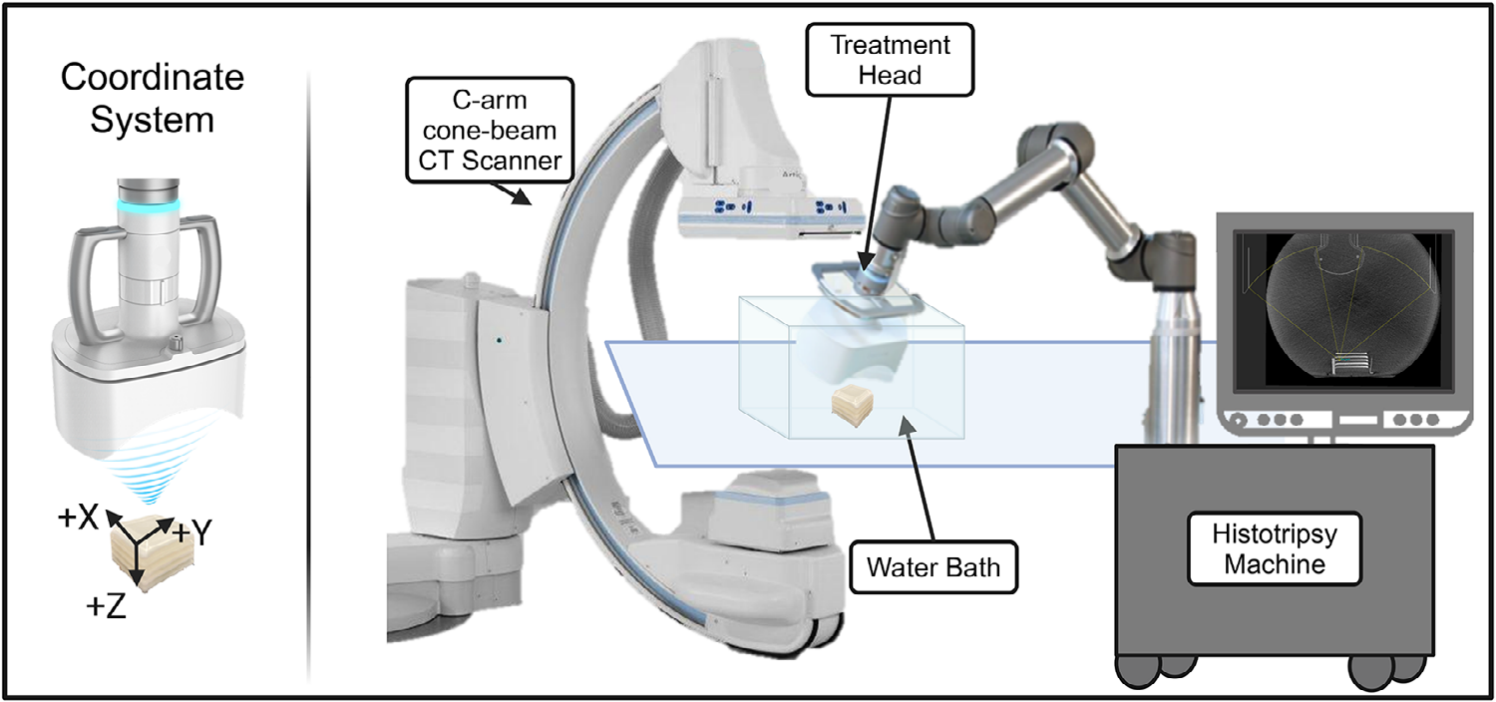
Experimental histotripsy setup. An illustration of the histotripsy coordinate system used in this study is presented on the left. The full experimental setup with the histotripsy machine positioned near and calibrated to the C‐arm can be seen on the right. The phantom is placed in the water bath for ultrasound coupling. The screen of the histotripsy machine provides an example of CBCT targeting with the CBCT image of the phantom and transducer location. Created in BioRender.^33^

Histotripsy treatments were performed with a 700 kHz multi‐element therapy transducer and system (Histosonics Inc, Plymouth, MN). An integrated, coaxially aligned diagnostic imaging probe, was present on the transducer but was not used in this study. Histotripsy targeting was performed using a floor‐mounted C‐arm of a clinical biplane system (Artis zee, Siemens Healthineers, Forchheim, Germany). Calibration of the robotic arm to the CBCT coordinate system was performed using a helical fiducial calibration phantom attached to the robotic arm.^30^ The phantoms were imaged and treated in a degassed water bath in the C‐arm's field of view. The pre‐treatment CBCT scan (20 s, 111 kV, 1908 mAs, 496 projections) was obtained and reconstructed using conventional filtered back projection (512 × 512 × 391 voxels, 0.47 mm/voxel). The reconstructed image was imported into a treatment planning software on the ImFusion framework (ImFusion GmbH, Munich, Germany), and the location of the target was chosen in each phantom (Figure 2). The specific robotic arm position for treatment was exported to the histotripsy machine and the treatment head automatically positioned to target the location. The experimental setup is illustrated in Figure 2.

### 2.3 Single bubble cloud localization algorithm

**FIGURE 3:**
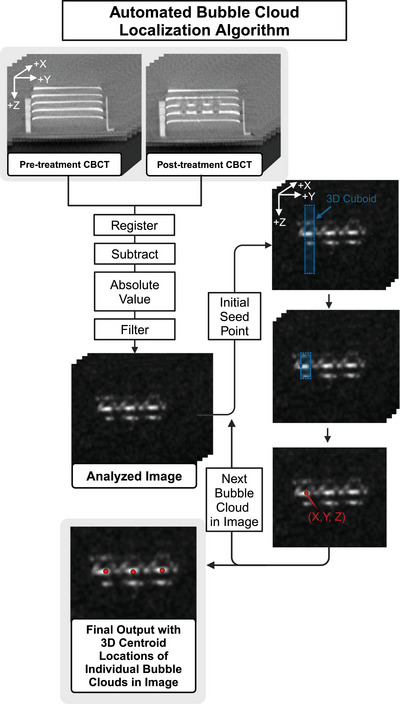
Illustration of the automated bubble cloud localization algorithm. The input of the algorithm is a pre‐treatment and post‐treatment CBCT image, and the output is 3D centroid locations for each bubble cloud in the image. This example illustrates a case where three bubble clouds are analyzed. Created in BioRender.^33^

To facilitate bubble cloud offset measurements, an algorithm was developed to automatically localize the bubble cloud centroid from pre‐ and post‐treatment CBCT image of the phantom (implemented in MATLAB, R2021a) (Figure 3). The pre‐ and post‐treatment CBCT images were manually cropped about the phantom, and rigidly registered (imregister) by minimizing the mean squared error (MSE) with a gradient descent optimizer, to account for any minor physical shifts of the phantom from the water bath. To identify the areas in the phantom where the bubble cloud caused mixing of the layers and thus a change in image intensity, the absolute difference between the two registered images was calculated. A gaussian filter (kernel size of 5 voxels and standard deviation of 1 voxel/0.47 mm) was applied to the subtraction image to improve convergence during the next steps. A 3D cuboid (4 mm × 4 mm × 30 mm) was placed over a seed point, which was manually chosen from the post‐treatment CBCT at the observed center of the bubble cloud treatment. The width of the cuboid was chosen to be slightly larger than that of the expected bubble cloud (3 mm), and the height of the cuboid ∼3 times that of the expected bubble cloud (7–10  mm) to encompass all possible treated layers. The mean intensity of the subtraction image within the cuboid was maximized using the Nelder‐Mead simplex approach (fminsearch) to find the region of interest (ROI) that best localized the treated layers.^34^ This was performed in two steps. First, the location of a cubical ROI in the plane parallel to the layers of the phantom was optimized. The optimization was performed multiple times (*n* = 7), where the initial seed point location was varied by two voxels in all directions and an extra four voxels in the direction perpendicular to the layers to further reduce the risk for local minima. In a second step, the top and bottom of the bubble cloud were identified along the *Z* direction (perpendicular to the layers). The middle third of the cuboid was identified and averaged in the *X* and *Y* directions to create a 2D, singular column vector. A pixel‐by‐pixel analysis was then done along the *Z* direction of this column vector starting both at the top and the bottom to find the first location that is greater than the mean intensity of the entire 2D vector. If the final distance between the identified top and bottom of the bubble cloud was at or greater than the expected bubble cloud height (8 mm), the center of the rectangle was used for the *Z* coordinate. If the distance was shorter than 8 mm (i.e., a case where the bubble cloud was located directly between two barium layers so the phantom mixing does not clearly identify the top and bottom of the bubble cloud) the intensity weighted centroid (first image moment) was used to determine the *Z* coordinate. After all three coordinates were automatically identified, the Euclidean distance between the calculated centroid and the planned centroid (assumed as the geometrical focal point of the transducer based on a computer aided design (CAD) model) was exported as the 3D bubble cloud offset. To ensure the automated localization algorithm was equivalent to manual segmentation, an experiment to compare manual versus automated segmentation was conducted with *n* = 12 phantoms. Bubble cloud treatment zones were manually segmented by a blinded reviewer using a grow‐cut algorithm (3D Slicer, 5.0.3).^35^ An equivalence test using lower bounds (Δ) of ±1.5 mm (half of the minimum bubble cloud width) was used.

### 2.4 Multi‐bubble cloud calibration approach

A calibration approach with multiple bubble clouds was designed to improve variability beyond a single bubble cloud treatment zone. To this end, a fixed treatment pattern consisting of four individual bubble cloud treatments was prescribed. The target locations for the bubble clouds were spaced 1 cm apart in the *Y* direction and 1 mm apart in the direction perpendicular to the phantom layers (*Z*‐direction) (see Figure 2 for axis orientation). This spacing was chosen to create the most amount of unique treatment positions (each barium layer is 3–4  mm apart). The bubble cloud localization algorithm found the centroids of each individual bubble cloud treatment zone (as described above) and the average of these centroids was calculated. This coordinate was used as the initial seed point for a new, second step of the algorithm which optimized the mean intensity within all treatment zones simultaneously. This approach takes advantage of the known distance between bubble clouds by forcing the ROIs for the cost function to have the same fixed distance apart. The same algorithmic steps of the single bubble cloud localization algorithm (Section 2.3, Figure 3) were used, except in this case, the mean intensity of all four treatment zones were optimized together, and the output was the average centroid of the four ROI locations representing the treated areas. The Euclidian distance between the average centroid of the treatment areas and the average centroid of the targeted areas was the reported 3D bubble cloud offset.

### 2.5 Analysis

#### 2.5.1 Effect of treatment duration and location on visibility

The phantom was constructed to visualize a single bubble cloud via mixing of high and low attenuating layers. It was hypothesized that a longer bubble cloud treatment duration would increase mixing and thus improve visibility on CBCT. Therefore, six phantoms were treated with bubble clouds of varying durations (5, 10, 15, 20, 25, and 30 s, *n* = 10 per group) and imaged using the fixed C‐arm. The treatment zone was targeted on a barium layer (*n* = 30) or between two barium layers (*n* = 30). Testing both locations was important to determine the visibility of the bubble cloud at varying *Z* locations. Bubble clouds were created in adjacent, independent areas of the phantom to ensure no overlap. Analysis of the visibility as a function of treatment time was done in MATLAB (R2021a) by manually placing an ellipse of the nominal bubble cloud size (4 × 4 × 8  mm) over the observed center of the mixed treatment zone on the post‐treatment CBCT image. The pre‐treatment and post‐treatment images were rigidly registered (as described in Section 2.3) and subtracted to obtain an image of the change that the bubble cloud destruction created. The absolute value of the subtraction image was used for the analysis so mixing of both layers of high and low attenuation could be detected. A gaussian filter (kernel size of five and standard deviation of one) was applied and the mean intensity within the ellipse was calculated for each trial as a measure of visibility.

To analyze the influence of treatment time on the change in image intensity, the treatment position datapoints were pooled (*n* = 10 per treatment time). The treatment duration with the greatest intensity was used for future experiments.

To analyze the influence of treatment position on change in image intensity, the influence of treatment time was removed by subtracting the average intensity of the group from each intensity measurement using Equation (1),

(1) $$A_{i,j,k}=\left(a_{i,j,k}-\frac{1}{2n}\;\sum_{k=0}^{1}\sum_{i=1}^{n}a_{i,j}\right)$$

where $a_{i,j,k}$ is the mean intensity difference in the treatment zone between the pre‐ and post‐treatment image for the *i*th trial of the *j*th treatment duration group. The index *k* is 0 if the treatment was between layers and 1 if it was on a layer. The parameter n is the total number of trials per treatment time and location (on vs. between layers). The differences in mean intensity within the ellipses were analyzed.

#### 2.5.2 Localization accuracy of the layered phantom

The accuracy of this calibration method is dependent on the minimum distance between planned and actual bubble cloud location that the phantom and the algorithm can resolve. To estimate this localization accuracy, 19 phantoms were treated with bubble cloud treatments with known distances apart and imaged using the Artis zee. Each direction (*X*, *Y*, *Z*) was tested independently by creating adjacent bubble clouds 1, 2, 3, 4, 5, and 6 mm apart. First, a reference bubble cloud was created. Then, the robot was translated the predefined distance, and a treatment was performed to create a new, adjacent bubble cloud. Each translation distance was replicated 10 times for each direction. The centroids of the treatment zones were automatically localized using the bubble cloud localization algorithm. The centroid distance between the reference and translated bubble cloud was compared to the planned translation in *X*, *Y*, and *Z* directions and the mean residual error was calculated using Equation (2),

(2) $$\text{Mean Residual Error}\left(MRE\right)=\frac{1}{6n}\sum_{j=1}^{6}\sum_{i=1}^{n}\left(d_{i,j}-j\right)$$

where $d_{i,j}$ is the distance from the reference cloud to the moved cloud measured with the automatic algorithm for the i th trial, j is the planned distance that was translated, and n is the number of trials per translation. The MRE and standard deviation between the measured and planned translations were reported.

#### 2.5.3 Robot to bubble cloud calibration

Since the bubble cloud calibration offset is unknown for each transducer, the algorithm needs to be robust and consistently locate the bubble cloud anywhere in the phantom (i.e., across many combinations of layers). To this end, a multi‐bubble cloud calibration approach was created which consisted of a bubble cloud pattern with four adjacent bubble clouds, 1 mm apart in Z and 10 mm apart in Y. Four phantoms were treated with the multi‐bubble cloud pattern and the variability between measuring the bubble cloud locations with a single (Section 2.3) versus multiple (Section 2.4) bubble cloud approach was analyzed. Assuming this robot to bubble cloud offset is consistent for the same transducer (at least within the same treatment session), the variability of the measurement approach can be characterized by the mean absolute deviation between experiments,

(3) $$\mathrm{Mean}\;\mathrm{Absolute}\;\mathrm{Deviation}\;\left(MAD\right)=\frac{1}{n}\;\sum_{i=1}^{n}\left|\;x_{i}-m\left(x\right)\right|$$

where n is the number of trials, $x_{i}$ is the offset in a particular direction, and m(x) is the mean offset in that direction, respectively for the n trials. MAD is calculated for each of the *X*, *Y*, and *Z* directions. Using the same phantoms for both approaches result in *n* = 16 for individual bubble clouds and *n* = 4 for the pattern calibration approach.

#### 2.5.4 Calibration offsets from multiple transducers

To test the algorithm, three more histotripsy transducers in addition to the transducer used in Section 2.5.3, were used to treat 18 phantoms (*n* = 6 per transducer). Using the same water bath set up, a fixed C‐arm, and the bubble cloud calibration pattern with four bubble clouds, the 3D offsets for each transducer were measured.

#### 2.5.5 Statistical analysis

For the experiments, a Shapiro–Wilk test was used to test for non‐normality in the data and if appropriate, a Levene's test was used to assess equality of variance. Then either a Kruskal–Wallis test with Dunn's post hoc comparison, a Mann–Whitney *U* test, or a student's *t*‐test for two independent samples were used depending on the number of groups and normality. For the localization accuracy experiment with two quantitative variables, normality was assumed via the Central Limit Theorem and a Pearson correlation coefficient was calculated. A significance level of *α* = 0.05 was used for all statistical tests.

## 3 RESULTS

### 3.1 Effect of treatment duration and location on visibility

**FIGURE 4:**
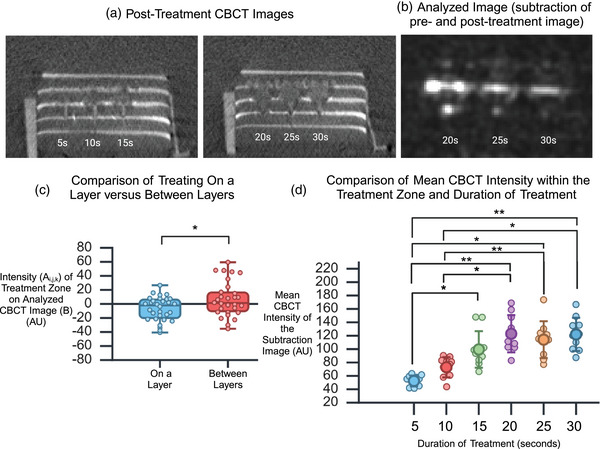
Bubble cloud treatment parameters (location and duration) were evaluated to create the most visible treatment. (a) Post‐treatment CBCT images show the bubble cloud treatments of varying durations (5–30 s). (b) Example of the image used to analyze the intensity versus treatment time. The analyzed image is the result of subtracting the pre‐ and post‐treatment CBCT scans, taking the absolute value, and applying a gaussian filter. Bright areas represent the layer mixing due to mechanical disruption from histotripsy treatment and therefore represent the bubble cloud location. (c) Box plot comparing the intensity of the treatment zone (*A_i,j,k_*) on the analyzed CBCT image for 30 trials (*i*) of treating on a layer (*k*) and 30 trials of treating between layers. (d) Box plot comparing the mean CBCT intensity within the treatment zone on the analyzed image (b) for different durations of treatment time (5, 10, 15, 20, 25, 30 s). Statistical significance between groups in plots (c) and (d) is denoted by * = *p* < 0.05, ** = *p* < 0.001. The error bars show 5–95th percentiles. Created in BioRender.^33^

The multilayer phantom with 11 alternating layers of plain agar and agar plus barium sulfate powder were easily visible in the water bath on CBCT imaging (Figure 1). Mixing of layers was observed in CBCT images after bubble cloud treatments of all durations (5–30 s) (Figure 4a). Thus, subtracting the pre‐ and post‐treatment CBCT images showed the observable change in intensity after the bubble cloud treatments (Figure 4b). Table 1 and Figure 4c show the output of Equation (1), representing intensity values of treating on a layer versus between a layer for each datapoint normalized by the mean of its treatment duration group. A Shapiro‐Wilk test did not show evidence of non‐normality (*W* = 0.971, *p* = 0.554 for treating on a layer and *W* = 0.948, *p* = 0.150 for between a layer). Also, Levene's test for equality of variances did not show evidence of homogeneity of variances (*p* = 0.1468). Therefore, a Student's *t*‐test for two independent samples was performed and showed that treating between layers had a statistically greater absolute intensity change from pre‐ and post‐treatment images (*M* = 6.8, SD = 24.5) compared to treating on a layer (*M* = −6.8, SD = 17.1), *t*(58) = 2.49, *p* = 0.016.

**TABLE 1:** Analysis of the effect of bubble cloud treatment duration (s) and location on visibility. Visibility is quantified by the greatest mean intensity within the treatment zone of the analyzed CBCT image (pre‐treatment minus post‐treatment image). Shapiro–Wilk test results were significant for non‐normality for the 15 s (denoted with an *). The table reports *A_i,j,k_* for each trial (*i*), treatment time (*j*), and treatment position (*k*) (Equation 1). *A_i,j,k_* is used to analyze differences between treating on a layer versus between layers.

	5 seconds	10 seconds	15 seconds	20 seconds	25 seconds	30 seconds
Mean intensity on analyzed CBCT image	52.5	72.9	99.5	122.9	114.1	122.0
Min–Max range	8.1	15.1	27.3	27.8	27.5	24.8
Shapiro–Wilk test result (W, *p*‐value)	0.896, 0.199	0.903, 0.237	0.842, 0.046*	0.949, 0.651	0.901, 0.190	0.974, 0.928

Position on/between layers	On	Between	On	Between	On	Between	On	Between	On	Between	On	Between
*A*_Trials 1‐5_	0.12	1.84	−13.02	16.76	−21.78	−1.63	−21.26	45.77	−22.69	0.68	−24.53	−35.44
	4.21	6.06	−0.62	9.34	−16.49	−6.69	−12.12	8.07	6.80	−0.21	−0.01	−22.60
	1.80	10.06	3.53	14.91	−33.97	−5.89	−3.62	−20.63	15.85	−0.71	26.66	−12.30
	−8.54	−11.58	7.89	−30.23	1.95	48.03	−15.24	35.62	−37.85	59.46	12.65	44.49
	8.20	−12.16	7.34	−15.91	−11.25	47.72	−40.80	24.23	−30.65	9.33	13.44	−2.3

Figure 4d shows a box plot of treatment time versus mean absolute intensity change within the ellipse placed on the analyzed image for each duration of treatment (*n* = 10 per treatment duration). The duration of 20 s had the greatest mean intensity, 122.9, followed by 30 s (122.0), 25 s (114.1), 15 s (99.5), 10 s (73.0) and 5 s (52.5) across 10 trials. The Shapiro‐Wilk test showed no evidence of non‐normality in all groups except 15s (see Table 1 for results) and the Kruskal–Wallis test found significant differences, H(5, *n* = 60) = 38.73, *p* < 0.001. Post hoc comparisons were conducted using Dunn's comparison test to find the difference in mean intensity, which was statistically significantly different when comparing the 5 s and the 15 s (*z* = 3.23, *p* = 0.16), 20 s (*z* = 4.70, *p* < 0.0001), 25 s (*z* = 4.22, *p* = 0.0003), and 30 s (*z* = 4.75, *p* < 0.001) groups. The Dunn's comparison test also found statistically significant differences in mean intensity between the 10 s and 20 s (*z* = 3.39, *p* = 0.009), 25 s (*z* = 2.919, *p* = 0.041), 30 s (*z* = 3.44, *p* = 0.008) groups. Because the 20 s bubble cloud treatment induced the greatest intensity change and hence most visible treatment, the layer phantom was treated with 20 s bubble clouds for future evaluation.

### 3.2 Localization accuracy of the layer phantom

**FIGURE 5:**
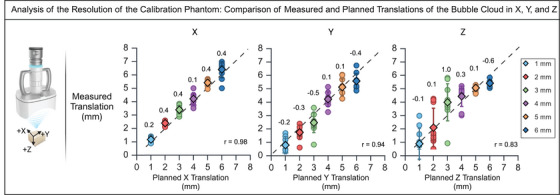
Analysis of the localization accuracy of the calibration phantom. Three strip plots show the measured translation (using the automatic bubble cloud localization algorithm) versus the planned translation in *X*, *Y*, and *Z* directions (coordinates relative to the transducer and phantom are shown in the upper left). The diamond represents the mean of the 10 trials for each planned translation. The difference between the diamond value and the planned translation for each movement is provided. A perfect agreement would place the diamond on the dashed diagonal line. The Pearson correlation coefficient (*r*) is labeled for each direction. If the diamond is above the dashed line, this represents an over‐estimation of the automated algorithm and the diamond below the line represents an under‐estimation of the planned translation. Created in BioRender.^33^

The difference between the automated and manual bubble cloud localization was significantly smaller than 1.5 mm all three directions (see Supplementary data for full analysis, Figure S1). The localization accuracy of the layer phantom and automated algorithm was determined by delivering a series of bubble cloud treatments at prescribed locations adjacent to each other. It was possible to create non‐overlapping, adjacent treatments in all directions and for all trials. Utilizing results from Section 3.1, each bubble cloud was treated with a 20 s duration. The reference bubble cloud was targeted on a barium layer to ensure consistency between trials, but the subsequent clouds resided anywhere in the phantom due to their planned translation. All automatically measured translations had a mean residual error of <0.3 mm in all three directions. The MRE and standard deviation was 0.3 ± 0.4 mm, −0.2 ± 0.6 mm and 0.1 ± 1.1 mm for the *X*, *Y*, and *Z* directions, respectively. The plots in Figure 5 illustrate the measured translation (difference between reference and translated cloud) versus the planned distance. A perfect match in measured and planned translation would place the points along the diagonal dashed line and a correlation coefficient of *r* = 1 (Figure 5). The measured translations all positively correlated with the planned movements, *r*(58) = 0.98, *p* < 0.001 for *X*, *r*(58) = 0.94, *p* < 0.001 for *Y*, *r*(58) = 0.83, *p* < 0.001 for *Z*. The *Z* direction had the greatest variability in measured translations for each planned translation but still showed relatively high correlation (*r* = 0.83) between the planned and average measured translation.

### 3.3 Robot to bubble cloud calibration

**FIGURE 6:**
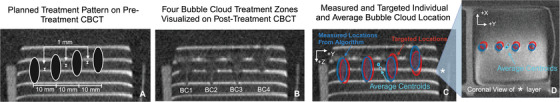
(a) Illustration of the planned multi‐bubble cloud treatment pattern outlined on the pre‐treatment CBCT image. (b) Post‐treatment CBCT image of the four bubble cloud treatments. (c and d) Post‐treatment CBCT image (C—axial, D—coronal) illustrating the bubble cloud location measured with the multi‐bubble cloud calibration approach (dark blue outline) and the targeted locations (red outline). The multi‐bubble cloud calibration approach compares the offset using the calculated average centroids (cyan). Created in BioRender.^33^

The phantom and multi‐bubble cloud calibration approach were used to measure the robot to bubble cloud calibration offset in four trials of a single transducer (Figure 6 and Table 2). The average robot to bubble cloud calibration offset for the tested transducer was −1.5 ± 0.1 mm, −0.8 ± 0.1 mm, and −1.5 ± 0.3 mm in *X*, *Y*, and *Z*, respectively, when analyzing the clouds in sets of four. The average calibration offset for the tested transducer when analyzing the 16 clouds individually were −1.5 ± 0.3 mm, 0.1 ± 1.3 mm, and −1.7 ± 1.5 mm in *X*, *Y*, and *Z*, respectively. Table 2 reports the offset measurements for the individual phantoms.

**TABLE 2:** Measured robot to bubble cloud offsets (difference in location between the average measured and targeted location) when using the multi‐bubble cloud calibration approach for a single transducer. The mean absolute deviation of the bubble cloud offset when measured one bubble cloud at a time (single, *n* = 16) or with multiple bubble clouds together (*n* = 4) is also presented. For the *Y* and *Z* directions, the multi‐bubble cloud calibration approach resulted in statistically significantly (*, *p* < 0.05) lower variability compared to analyzing the bubble clouds individually.

	Offsets measured using the multi‐bubble cloud calibration approach					Mean absolute deviation of offsets when measured with the single and multi‐bubble cloud approach		
Transducer axis	Phantoms treated				Average offset ± standard deviation	Individual bubble cloud (*n* = 16)	Multi‐bubble cloud (*n* = 4)	
	1	2	3	4				*p* Value
*X* (mm)	−1.4	−1.5	−1.5	−1.7	−1.5 ± 0.1	0.2 ± 0.1	0.1 ± 0.1	0.447
*Y* (mm)	−0.9	−0.9	−0.9	−0.7	−0.8 ± 0.1	1.1 ± 0.7	0.0 ± 0.0*	0.003
*Z* (mm)	−1.9	−1.6	−1.2	−1.4	−1.5 ± 0.3	1.2 ± 1.0	0.2 ± 0.1*	0.020

When using the single bubble cloud localization algorithm on each individual bubble cloud, the mean absolute deviation was 0.2, 1.1, and 1.2 mm in *X*, *Y*, and *Z*, respectively. When analyzing the bubble clouds together via the multi‐bubble cloud calibration approach, the MAD was 0.1, 0.0, and 0.2 mm (Table 2). The Shapiro Wilk test showed non‐normality for all groups with an average *W* = 0.76 and average *p* value of 0.024. Therefore, the Mann–Whitney *U* test was performed to evaluate whether the MAD significantly improved when performing the multi‐bubble cloud approach. The results indicated that using the multi‐calibration calibration approach had a significantly lower MAD than using a single bubble cloud approach in *Y* (*z* = −2.99, *p* = 0.003) and *Z* (*z* = −2.32, *p* = 0.020). In the *X* direction, however, there was not a significant difference in MAD (*z* = −0.76, *p* = 0.447) with either approach (Table 2).

### 3.4 Offsets measured from multiple transducers

**FIGURE 7:**
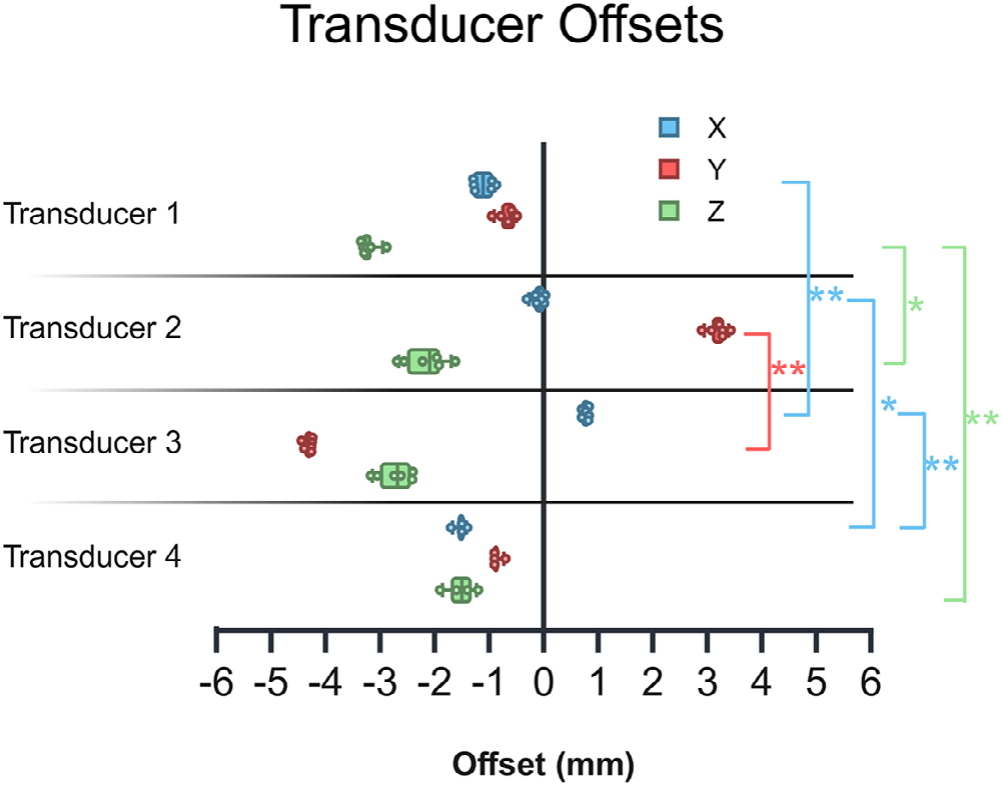
Transducer offsets measured in four different histotripsy transducers. Offsets were measured using the multi‐bubble cloud calibration approach. Statistical significance between transducers is denoted by * = *p* < 0.05, ** = *p* < 0.001. The error bars show 5–95th percentiles. Created in BioRender.^33^

Each transducer had a unique 3D transducer offset and had low variability between each phantom trial (Figure 7). The offset in Transducer 1 had a median and interquartile range (median (IQR)) of −1.1 (0.3) mm in *X*, −0.6 (0.2) mm in *Y*, and −3.3 (−0.1) mm in *Z*. Across the six trials, the offset in Transducer 2 was −0.1 (0.1) mm in *X*, 3.3 (0.2) mm in *Y*, and −2.1 (0.5) mm in *Z* whereas the offset in Transducer 3 was 0.8 (0.1) mm in *X*, −4.3 (0.1) mm in *Y*, and −2.7 (0.5) mm in *Z*. Finally, the offsets in Transducer 4 were −1.5 (0.1) mm in *X*, −0.9 (0.0) mm in *Y* and −1.5 (0.3) mm in *Z*. A Shapiro–Wilk test for all three directions (groups were normalized to their mean), showed evidence of non‐normality (*W* = 0.6248, *p* < 0.05). The Kruskal–Wallis test was performed to determine if there were differences in offsets across the four transducers. It showed a statistically significant difference between groups in *X* (*χ* ^2^(3) = 19.67, *p* = 0.0002), *Y* (*χ* ^2^(3) = 18.76, *p* = 0.0003), *Z* (*χ* ^2^(3) = 17.39, *p* = 0.0006). Post‐hoc comparisons using Dunn's test revealed a significant difference between Transducer 1–3, 2–4, and 3–4 in *X* (*p* = 0.007, 0.04, 0.0003, respectively), Transducer 2–3 in *Y* (*p* = 0.0001), and Transducer 1–2, and 1–4 in *Z* (*p* = 0.02, 0.0006, respectively).

## 4 DISCUSSION

This work presents a histotripsy robot to bubble cloud calibration method with a layered phantom that allows for visualization and automated localization of single bubble cloud treatment zones from CBCT images. An automated multi bubble cloud calibration approach was presented to accurately calibrate the histotripsy robot to the bubble cloud—a crucial development for increasing accuracy for CBCT guided histotripsy treatments.

The multi‐layer phantom was created with layers ∼3 mm apart to allow for mixing of high and low attenuating areas during treatment. Both layers were thinner than previously reported phantoms to allow for single bubble cloud detection.^29^, ^31^ The extent of layer mixing showed a dependence on the duration of the bubble cloud treatment. Bubble cloud treatments with a 20 s duration yielded the greatest intensity change on CBCT, and thus were the most visible treatment zones compared to other durations evaluated and was used for further testing. In the post‐treatment CBCT images of 20 s or greater, there was more cavitation along the beam path that extended the treatment zone toward the transducer. The manual and automatic algorithms were made robust to this by weighting all Z positions equally: the manual method used a bounding box to determine the centroid, and the automatic algorithm uses a cuboid shape to optimize the mean intensity. Importantly, the automatic localization algorithm, a faster approach with reduced user bias, was found to identify the centroid of the single bubble cloud treatment zone just as well as the manual segmentation approach could, within a 1.5 mm margin (see Supplementary data, Figure S1).

The localization accuracy of the phantom was estimated by evaluating the mean residual error of planned versus measured translations of the bubble cloud. In the phantom, this was found to be on average < 0.3 mm in all directions and across all six translations, suggesting that the phantom and algorithm can resolve sub‐millimeter translations in location. The largest variability of measured translation per planned translation (specifically for 1–3 mm) was seen in the *Z* direction (perpendicular to the barium layers) when the planned translations were less than the distance between each layer (Figure 5). This is likely due to the barium sulfate powder seeding cavitation by decreasing the surface tension of the microscale gas bubbles in the agar and blurring the top and bottom of the bubble cloud treatment zone.^36^ Cavitation at barium layers is likely also a reason for the observed increase in mean intensity change when treating between layers versus on a layer. The multi‐bubble cloud pattern approach with bubble clouds that incorporate different amounts of layers, was developed to overcome this variability that may shift the location of the automatically identified bubble cloud. By forcing the algorithm to optimize the location of all four bubble clouds simultaneously, the algorithm takes prior knowledge about the translation between individual bubble clouds into account and the results were more consistent (the mean absolute deviation was less in all three directions). The bubble cloud pattern is simple and can be implemented in less than 2 min, facilitating a fast and robust calibration that can be seamlessly integrated into the conventional histotripsy workflow. This method was further tested by comparing the transducer offsets from four different histotripsy transducers. There was very low variability in offset results within 4–6 phantoms in all three directions. In addition, there was at least one direction in each transducer that was statistically significantly different from each other. These results suggest that the algorithm is measuring distinct offsets of the transducer, and that each transducer is different. Authors suggest that until more complete studies of the origin of these offsets, that the calibration method be performed before any CBCT‐guided histotripsy procedures in the future.

There are limitations of this study. There was some variability in the layers when manufacturing the phantoms. Careful attention must be used to create the layers evenly and thinly. The algorithm was made robust to this by having two methods to determine the *Z* coordinate of the bubble cloud depending on how many layers the bubble cloud traversed (top minus bottom or the image moment). In addition, the multi‐bubble cloud approach purposefully positioned the clouds at different locations in *Z* direction to overcome any manufacturing variability that may affect an area of the phantom. In the localization accuracy analysis, the prescribed translations were defined in the robot coordinate system rather than the CBCT coordinate system. These coordinate systems were assumed to only differ by some translation, so effort was made to ensure perpendicular alignment of the histotripsy cart with the CBCT treatment table (Figure 2). That said, any millimeter translations of the cart or inconsistencies in leveling of the floor may induce deviations from purely lateral movement in CBCT coordinates. To resolve this limitation, the final calibration approach created a target in CBCT space, rather than histotripsy robot coordinates, for each of the four bubble clouds. In addition, accuracy of this method may depend on image quality and resolution of the c‐arm, and only a fixed c‐arm was tested in this study, with is a limitation.

This fast and reliable calibration method can be used for routine transducer calibration to increase accuracy of CBCT‐guided histotripsy treatments but also quantify any changes in transducer offsets that may occur from day‐to‐day use (e.g., detect damage of the transducer or estimate how frequently this calibration should be performed). The transducer offsets quantified here were measured in a single day, and future work will include tracking changes over time.

## 5 CONCLUSION

This work presents a calibration correction for registering the therapeutic focal point with CBCT to allow for precise and accurate CBCT guided histotripsy treatments. It includes the construction of an agar‐based histotripsy phantom with alternating high and low attenuating layers that mix from mechanical disintegration of histotripsy to allow for visualization of a single bubble cloud treatment zone with CBCT. Treating and analyzing four adjacent bubble clouds together produced more accurate and reproducible bubble cloud offset measurements than analyzing individual bubble clouds. The presented histotripsy bubble cloud calibration method (phantom and multi‐bubble cloud calibration approach) is automated, accurate, fast, and can be easily integrated in the current histotripsy workflow to bring CBCT guided histotripsy one step closer to being used with patients.

## CONFLICT OF INTEREST STATEMENT

Author K.F. is a consultant with HistoSonics, Inc. P.L. is a consultant and stockholder with HistoSonics, Inc. and receives research support from HistoSonics, a consultant with NeuWave/Ethicon, Inc., and receives research support from Siemens Healthineers. Author F.L. is a consultant, stockholder, receives research support, and is on the board of directors at HistoSonics, Inc., is a consultant with Ethicon, Inc., is on the Canon Medical Advisory Board, and has patents and royalties with Medtronic, Inc. Author T.Z. is a consultant, stockholder, receives research support from HistoSonics, Inc. and is a consultant with Ethicon, Inc.). Author M.W. is a consultant and receives research support from HistoSonics, Inc. Author M.S. receives research support from Siemens Healthineers. Authors G.M. and O.G.Z. do not have anything to disclose.

## Supporting information



Supporting information

Supporting information

## Data Availability

Data is available upon request.
