# Supplementary material for: Calibration correction to improve registration during cone‐beam CT guided histotripsy
Source: Med Phys. 2025 Jan 26;52(5):3216–27. doi: 10.1002/mp.17644 (PMC12059542; doi:10.1002/mp.17644)
Supplement: Supplementary file 1 — Supporting information [file MP-52-3216-s002.docx]

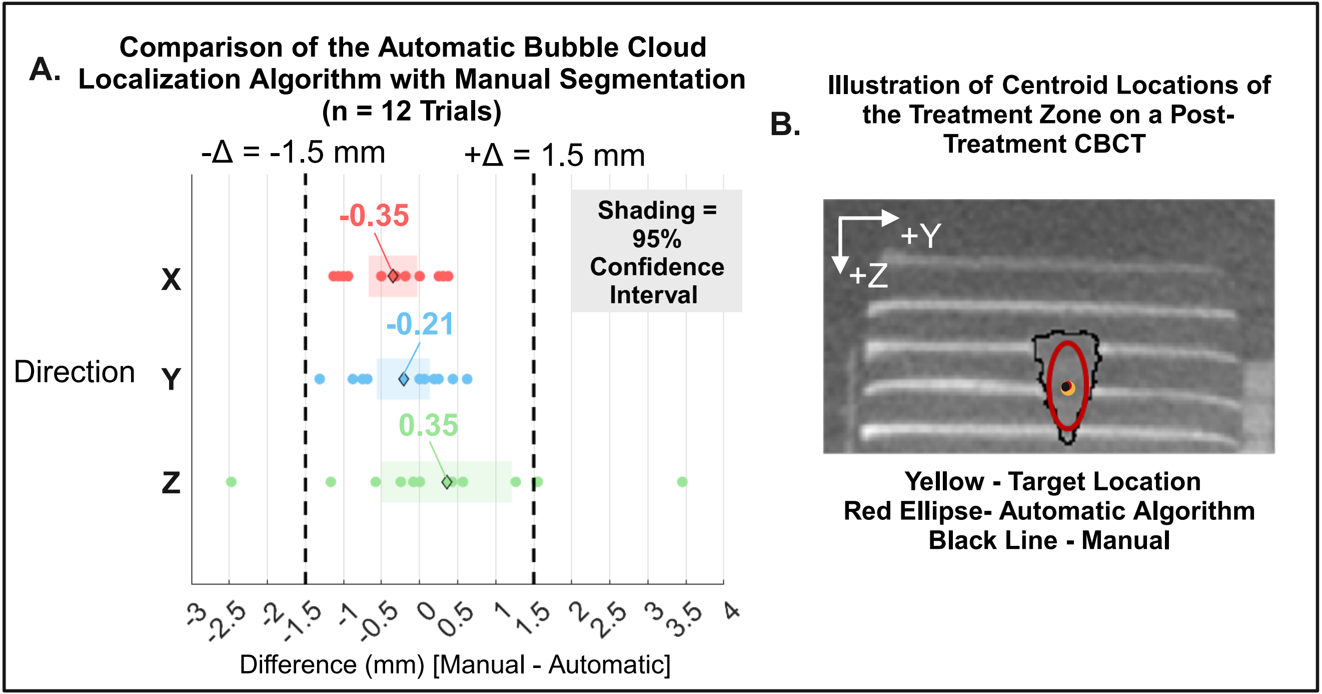

A.
Comparison of the Automatic Bubble Cloud Localization Algorithm with Manual Segmentation (n = 12 Trials)
-Δ = -1.5 mm
+Δ = 1.5 mm
Shading = 95% Confidence Interval
-0.35
-0.21
0.35
Direction
X
Y
Z
-3 -2.5 -2 -1.5 -1 -0.5 0 0.5 1 1.5 2 2.5 3 3.5 4
Difference (mm) [Manual - Automatic]
B.
Illustration of Centroid Locations of the Treatment Zone on a Post-Treatment CBCT
+Y
+Z
Yellow - Target Location
Red Ellipse- Automatic Algorithm
Black Line - Manual
