# Supplementary material for: Calibration correction to improve registration during cone‐beam CT guided histotripsy
Source: Med Phys. 2025 Jan 26;52(5):3216–27. doi: 10.1002/mp.17644 (PMC12059542; doi:10.1002/mp.17644)
Supplement: Supplementary file 2 — Supporting information [file MP-52-3216-s001.docx]

Supplementary Material

**Comparison of the automated bubble cloud localization algorithm with manual segmentation**

**Methods:**

Single bubble cloud treatment zones in 12 phantoms were used to compare the bubble cloud localization algorithm to manual segmentation. One bubble cloud was treated per phantom using the experimentally determined treatment duration (section 2.5.1) to maximize visibility. A reviewer, blinded to the automated bubble cloud localization results, manually segmented the bubble cloud treatment from the post-treatment CBCT image (acquired with a mobile CBCT scanner, CIOS, Siemens Healthineers, Forchheim, Germany, 30s, 111 kV, 528 mAs, 400 projections and reconstruction of 512 x 512 x 512, 0.33 mm/voxel) using a modified version of the grow-cut algorithm (3D Slicer, 5.0.3)^5^. The 3D centroid of a bounding box around the manually segmented region was determined. The single bubble cloud localization algorithm (as described in section 2.3) was used to calculate the centroid for comparison. The Shapiro-Wilk test was used to determine normality of the difference between the methods, and an equivalence test using upper and lower bounds (Δ) of ±1.5 mm (half of the minimum bubble cloud width). A significance level of α = 0.05 was used for all statistical tests.

**Results:**

The average differences between manual and automatically extracted bubble cloud centroids were -0.35 ± 0.6 mm, -0.21 ± 0.6 mm and 0.35 ± 1.5 mm in the X, Y, and Z directions, respectively (n=12, Figure 1A). The Shapiro-Wilk test did not show evidence of non-normality in all three directions (W = 0.897, 0.915, 0.961; p = 0.143, 0.248, 0.704 for X, Y and Z directions, respectively). Based on an equivalence test with an equivalence range of d = -1.5 to 1.5mm (p = <0.001, <0.001 and 0.024 for X, Y and Z, respectively), there is the absence of meaningful difference in all three directions between measuring the bubble cloud location with a manual and automatic approach (Figure 1A). Figure 1B illustrates an example of the manual segmentation results (black outline and centroid), the calculated centroid from the bubble cloud localization algorithm (ellipse and red dot) and the target location (yellow dot).

**
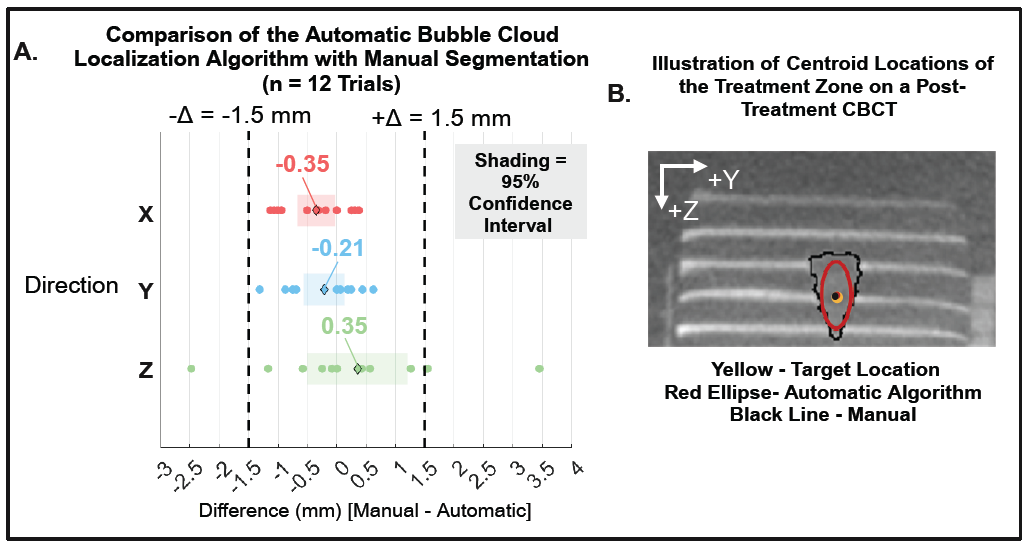
**

Figure S-1: Comparison of the bubble cloud treatment zone centroid when calculated from a manual segmentation and the automated algorithm. The plot (A) shows the difference in centroid location in 12 trials along the X, Y, and Z directions. The 95% confidence intervals (shaded regions) all lie within the 1.5 mm equivalence bounds. The mean of the trials is provided and marked with a diamond. An example case (B) shows differences between the segmentations (outlines) and centroids (dots) calculated manually (black) and automatically (red). The yellow dot represents the targeted location.
